# Supplementary figures and images for: Complexes of vertebrate TMC1/2 and CIB2/3 proteins form hair-cell mechanotransduction cation channels
Source: eLife. 2025 Jan 8;12:RP89719. doi: 10.7554/eLife.89719 (PMC11709434; doi:10.7554/eLife.89719)

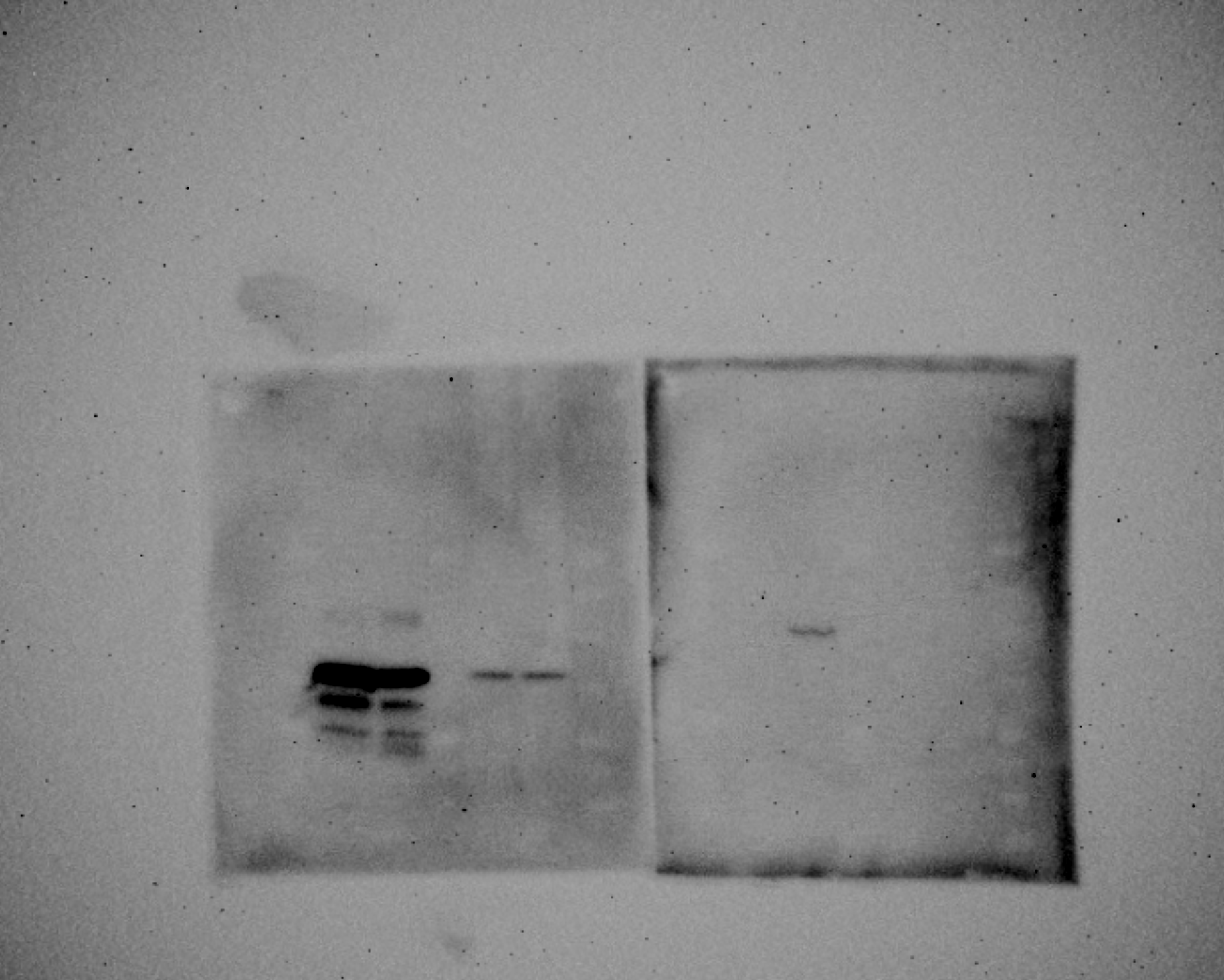

Supplement: Figure 1—source data 1. [file elife-89719-fig1-data1.zip › Figure1F-Ahmed lab 2020-02-06 15h19m23s(Chemiluminescence).tif]

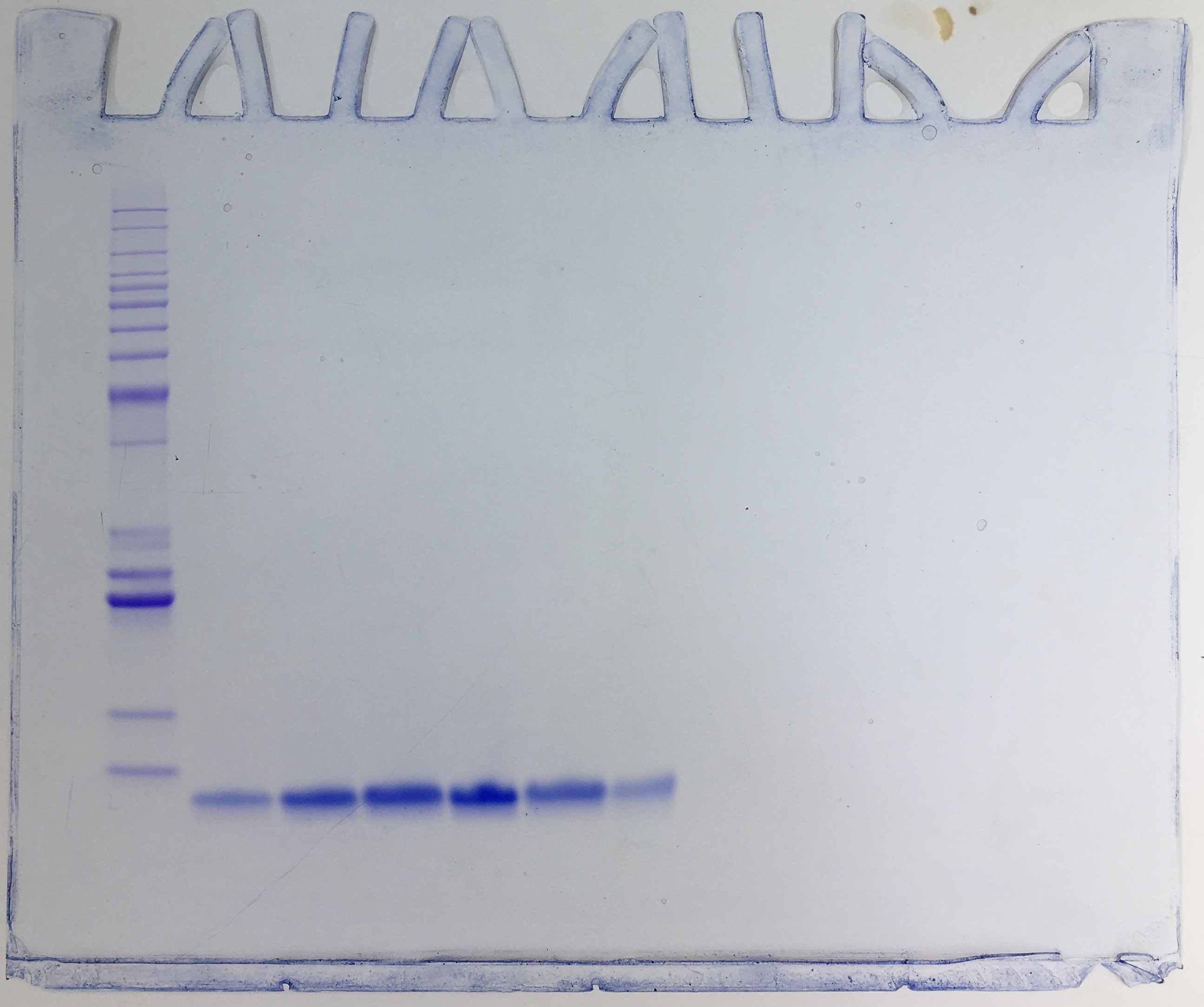

Supplement: Figure 7—figure supplement 5—source data 1. [file elife-89719-fig7-figsupp5-data1.zip › 2021-03-06 (hsTMC1MIN).jpg]

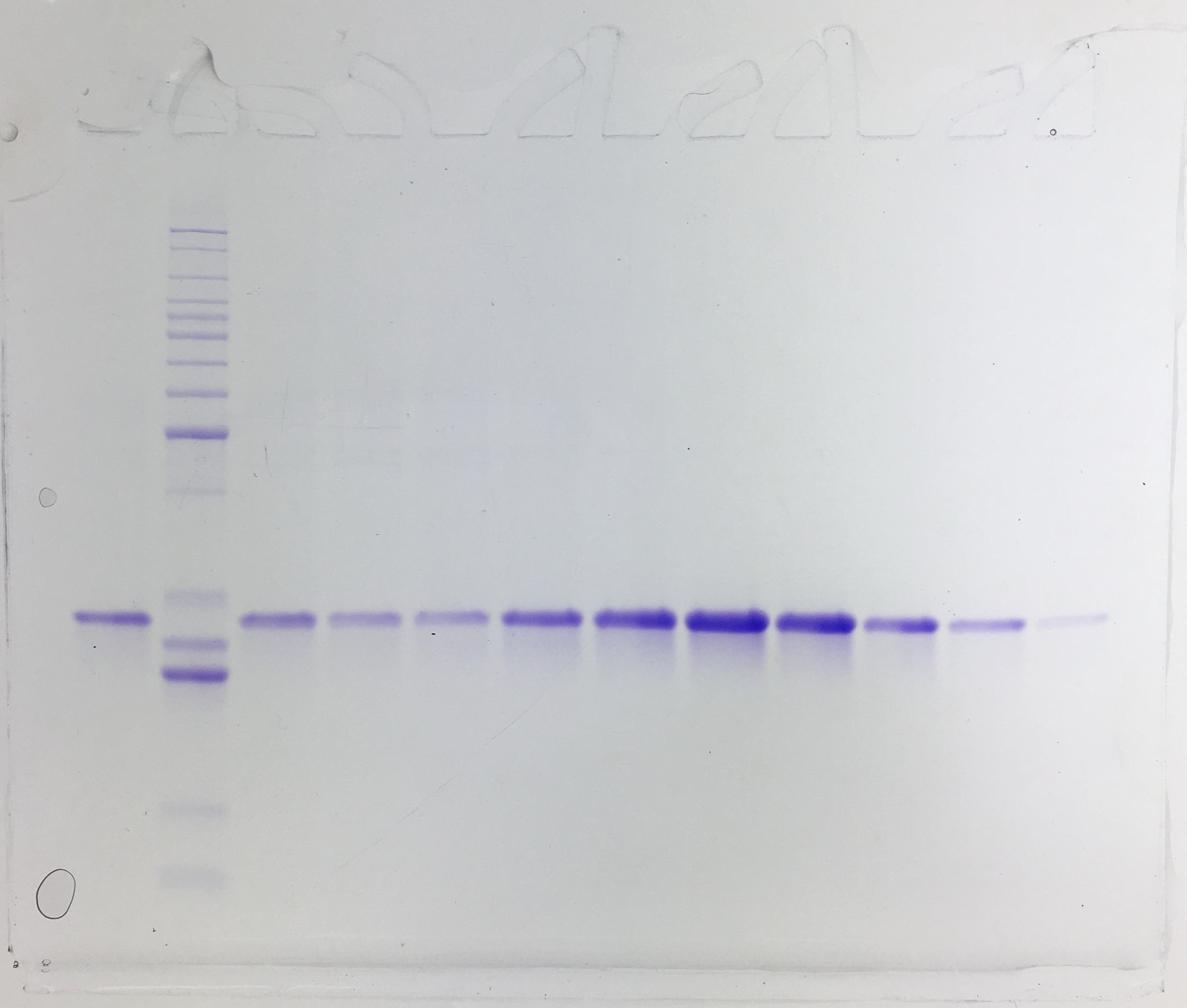

Supplement: Figure 7—figure supplement 5—source data 1. [file elife-89719-fig7-figsupp5-data1.zip › 2021-12-09-2 (hsCIB2KozDel).jpg]

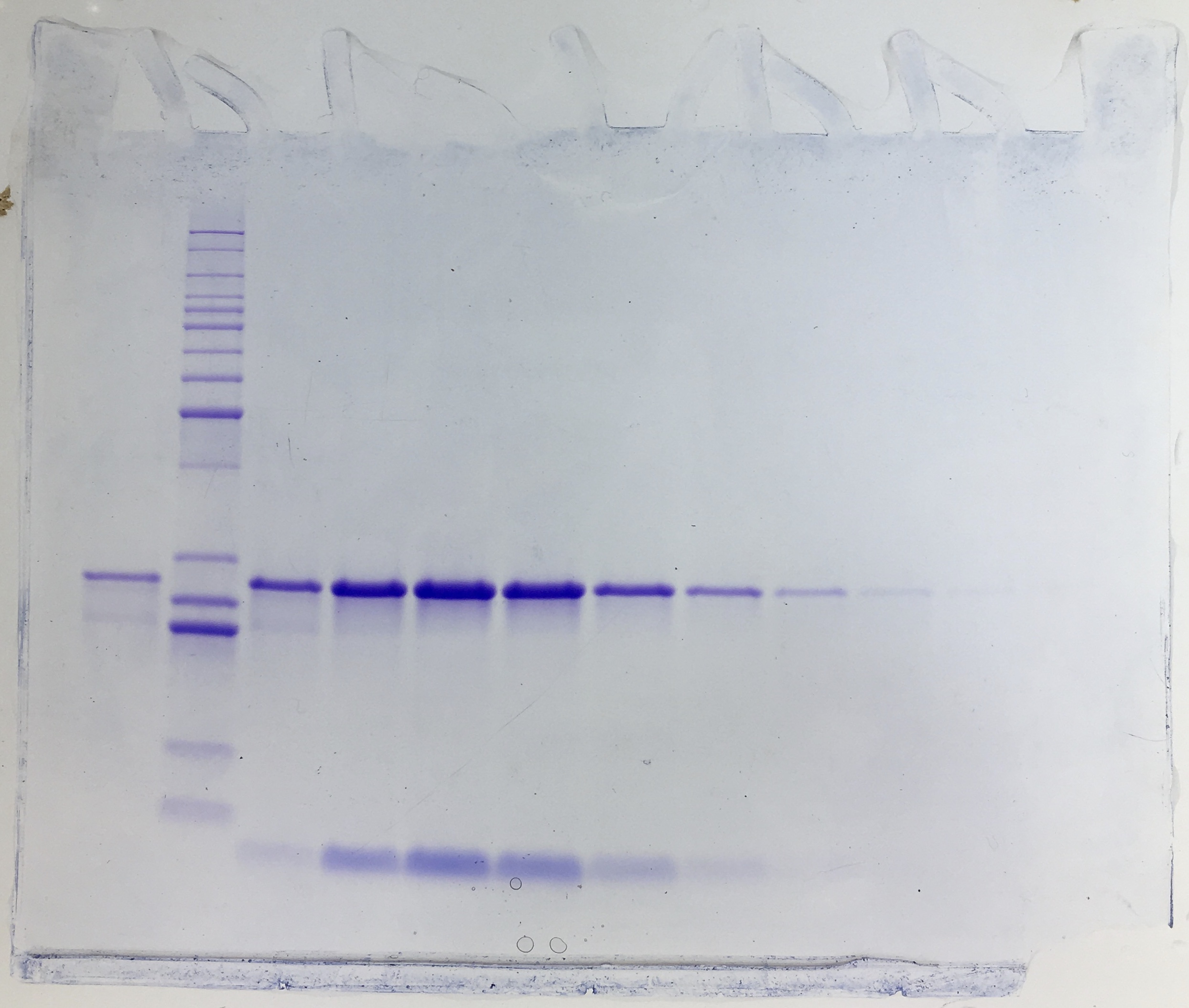

Supplement: Figure 7—figure supplement 5—source data 1. [file elife-89719-fig7-figsupp5-data1.zip › 2022-03-21-1 (hsCIB2KozDel_hsTMC1IL1).jpg]

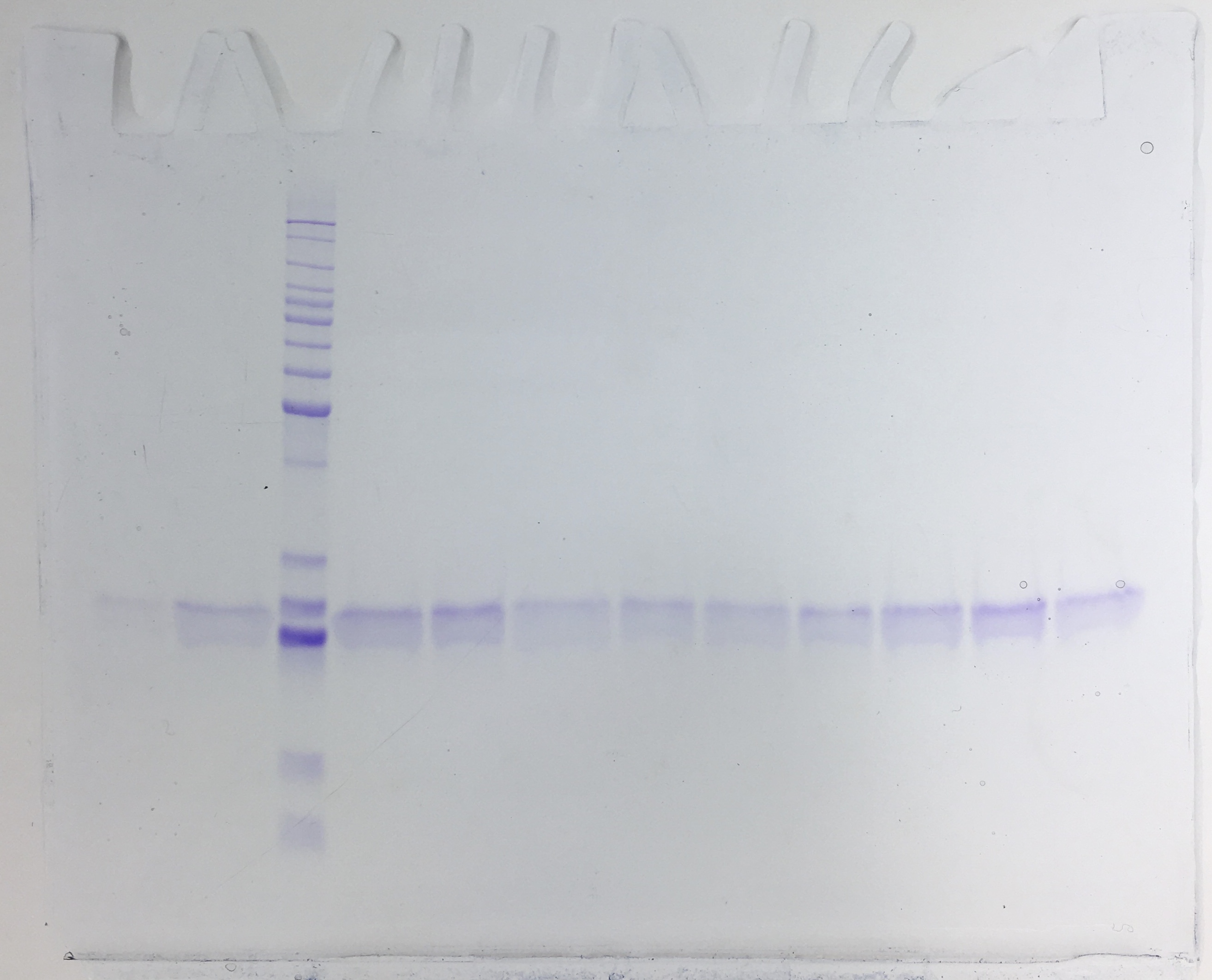

Supplement: Figure 7—figure supplement 5—source data 1. [file elife-89719-fig7-figsupp5-data1.zip › 2022-04-18-2 (15N-hsCIB3KozDel).jpg]

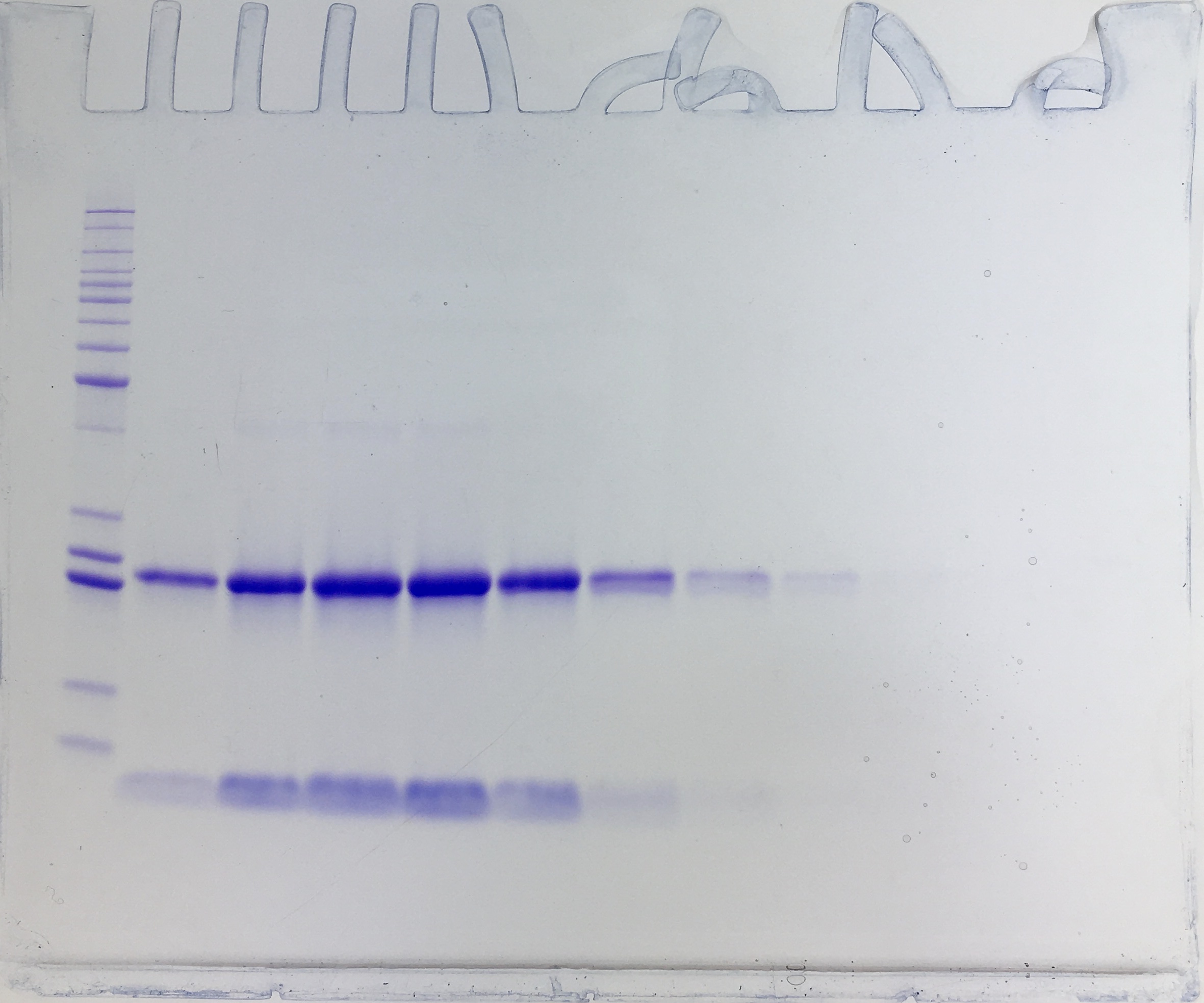

Supplement: Figure 7—figure supplement 5—source data 1. [file elife-89719-fig7-figsupp5-data1.zip › 2022-04-21-1 (15N-hsCIB3KozDel_hsTMC1IL1).jpg]
