# Supplementary material for: Complexes of vertebrate TMC1/2 and CIB2/3 proteins form hair-cell mechanotransduction cation channels
Source: eLife. 2025 Jan 8;12:RP89719. doi: 10.7554/eLife.89719 (PMC11709434; doi:10.7554/eLife.89719)
Supplement: Figure 8—source data 1. [file elife-89719-fig8-data1.pdf]

Figure 8—source data 1. Summary of simulations

System	Label	Ca^{2+}	Membrane	Start	Length (ns)	Voltage (V)	Size (# of atoms)	Initial Size (nm^3)
	S1a			-	100	0†		
	S1b			S1a	100	-0.5		
	S1c			S1b	100	-0.25		
hs TMC1 + *hs* CIB2	S1d	Yes	POPC	S1a	960	0	395,283	16.1 x 16.1 x 16.6
	S1e			S1d	480	-0.5		
	S1f			S1e	480	-0.25		
	S1g			S1f	960	-0.125		
	S2a			-	100	0†		
	S2b			S2a	100	-0.5		
	S2c			S2b	100	-0.25		
hs TMC1 + *hs* CIB2	S2d	No	POPC	S2a	960	0	395,200	16.1 x 16.1 x 16.6
	S2e			S2d	480	-0.5		
	S2f			S2e	480	-0.25		
	S2g			S2f	960	-0.125		
	S3a			-	100	0†		
	S3b			S3a	99.25	-0.5		
	S3c			S3b	100.875	-0.25		
hs TMC1	S3d	-	POPC	S3a	960	0	395,146	16.1 x 16.1 x 16.6
	S3e			S3d	480	-0.5		
	S3f			S3e	480	-0.25		
	S3g			S3f	960	-0.125		
	S4a			-	100	0†		
	S4b			S4a	100	-0.5		
hs TMC1 + *hs* CIB2	S4c	Yes	Mixed[a]	S4a	240	0	425,170	16.8 x 16.7 x 16.6
	S4d			S4c	480	-0.43		
	S5e			S4c	720	0		
hs TMC1 + *hs* CIB2	S5a	No	Mixed[a]	-	100	0†	424,916	16.8 x 16.7 x 16.6
	S5b			S5a	100	-0.5		
hs TMC1	S6a	-	Mixed[a]	-	100	0†	425,224	16.8 x 16.8 x 16.6
	S6b			S6a	100	-0.5		
hs TMC1 + *hs* CIB3	S7a	Yes	POPC	-	100	0†	394,843	16.1 x 16.1 x 16.6
	S7b			S7a	100	-0.5		
	S8a			-	100	0†		
hs TMC1 + *hs* CIB3	S8b	No	POPC	S8a	100	-0.5	394,750	16.1 x 16.1 x 16.6
	S8c			S8b	100	-0.25		
hs TMC1 + *hs* CIB3	S9a	Yes	Mixed[a]	-	100	0†	424,929	16.8 x 16.8 x 16.6
	S9b			S9a	100	-0.5		
hs TMC1 + *hs* CIB3	S10a	No	Mixed[a]	-	100	0†	425,005	16.8 x 16.7 x 16.6
	S10b			S10a	100	-0.5		
hs TMC2 + *hs* CIB2	S11a	Yes	POPC	-	100	0†	434,265	16.8 x 16.9 x 16.7
	S11b			S11a	100	-0.5		
hs TMC2 + *hs* CIB2	S12a	No	POPC	-	100	0†	433,630	16.9 x 16.9 x 16.6
	S12b			S12a	100	-0.5		
hs TMC2	S13a	-	POPC	-	100	0†	433,327	16.9 x 16.9 x 16.7
	S13b			S13a	100	-0.5		
hs TMC2 + *hs* CIB2	S14a	Yes	Mixed[a]	-	100	0†	427,357	16.8 x 16.8 x 16.5
	S14b			S14a	100	-0.5		
hs TMC2 + *hs* CIB2	S15a	No	Mixed[a]	-	100	0†	427,254	16.8 x 16.8 x 16.5
	S15b			S15a	100	-0.5		
hs TMC2	S16a	-	Mixed[a]	-	100	0†	428,031	16.8 x 16.8 x 16.6
	S16b			S16a	100	-0.5		
hs TMC2 + *hs* CIB3	S17a	Yes	POPC	-	100	0†	429,479	16.8 x 16.8 x 16.6
	S17b			S17a	100	-0.5		
hs TMC2 + *hs* CIB3	S18a	No	POPC	-	100	0†	429,600	16.9 x 16.8 x 16.6
	S18b			S18a	100	-0.5		
hs TMC2 + *hs* CIB3	S19a	Yes	Mixed[a]	-	100	0†	428,972	16.8 x 16.8 x 16.6
	S19b			S19a	100	-0.5		
hs TMC2 + *hs* CIB3	S20a	No	Mixed[a]	-	100	0†	428,904	16.8 x 16.9 x 16.6
	S20b			S20a	100	-0.5		
				Total time	14,480.125			

a lipid membrane bilayer was comprised of 65% POPC, 30% cholesterol and 5% PIP2. † denotes equilibrium simulations that consisted of 2,000 steps of minimization, 0.5 ns of dynamics with everything excluding lipid tails fixed/constrained, 0.5 ns of dynamics with harmonic constraints applied to the protein (1 kcal mol^{-1} Å^{-2}), 1 ns of free dynamics in the *NpT* ensemble (γ = 1 ps^{-1}), and up to 100 ns of free dynamics in the *NpT* ensemble (γ = 0.1 ps^{-1}).
