# Supplementary material for: Complexes of vertebrate TMC1/2 and CIB2/3 proteins form hair-cell mechanotransduction cation channels
Source: eLife. 2025 Jan 8;12:RP89719. doi: 10.7554/eLife.89719 (PMC11709434; doi:10.7554/eLife.89719)
Supplement: Figure 8—source data 2. [file elife-89719-fig8-data2.pdf]

Figure 8—source data 2. Ion conduction									
Label	Length (ns)	Voltage (V)	K^+ (A)	K^+ (B)	Cl^- (A)	Cl^- (B)	$I^†$ (pA)	$C^†$ (pS)	BSA (Å^2)
S1a	100	0	-	-	-	-	-	-	5,989.7
S1b	100	-0.5	1	38	0	0	62.4	124.8	5,595.7
S1c	100	-0.25	3	21	0	0	38.4	153.6	-
S1d	960	0	-	-	-	-	-	-	4,876.7
S1e	480	-0.5	274	125	17	0	138.7	277.3	4,755.1
S1f	480	-0.25	92	55	1	0	49.3	197.3	-
S1g	960	-0.125	51	37	0	0	14.7	117.3	-
S2a	100	0	-	-	-	-	-	-	5,661.8
S2b	100	-0.5	7	23	0	1	49.6	99.2	5,922.4
S2c	100	-0.25	3	16	0	0	30.4	121.6	-
S2d	960	0	-	-	-	-	-	-	5,467.1
S2e	480	-0.5	303	183	6	2	164.7	329.3	5,385.7
S2f	480	-0.25	118	151	1	1	90.3	361.3	-
S2g	960	-0.125	1	58	0	0	9.8	78.7	-
S3a	100	0	-	-	-	-	-	-	-
S3b	99.25	-0.50	0	39	0	0	62.9	125.7	-
S3c	100.875	-0.25	1	3	0	0	6.3	25.4	-
S3d	960	0	-	-	-	-	-	-	-
S3e	480	-0.5	41	24	0	0	21.7	43.4	-
S3f	480	-0.25	8	8	0	0	5.3	21.3	-
S3g	960	-0.125	3	9	0	0	2.0	16.0	-
S4a	100	0	-	-	-	-	-	-	5,903.7
S4b	100	-0.50	0	0	0	0	0.0	0.0	5,624.1
S4c	240	0	-	-	-	-	-	-	5,084.2
S4d	480	-0.43	8	0	0	0	2.7	6.2	4,966.0
S4e	720	0	-	-	-	-	-	-	5,039.4
S5a	100	0	-	-	-	-	-	-	5,553.5
S5b	100	-0.50	7	0	0	0	11.2	22.4	5,530.2
S6a	100	0	-	-	-	-	-	-	-
S6b	100	-0.50	3	0	0	0	4.8	9.6	-
S7a	100	0	-	-	-	-	-	-	5,993.6
S7b	100	-0.50	3	5	0	0	12.8	25.6	5,912.3
S8a	100	0	-	-	-	-	-	-	5,862.1
S8b	100	-0.50	43	16	0	0	94.4	188.8	6,069.5
S8c	100	-0.25	6	11	0	0	27.2	108.8	-
S9a	100	0	-	-	-	-	-	-	5,977.6
S9b	100	-0.50	3	2	0	0	8.0	16.0	5,806.4
S10a	100	0	-	-	-	-	-	-	6,022.6
S10b	100	-0.50	0	7	0	0	11.2	22.4	6,121.8
S11a	100	0	-	-	-	-	-	-	5,793.6
S11b	100	-0.50	1	0	0	0	1.6	3.2	5,718.4
S12a	100	0	-	-	-	-	-	-	5,550.7
S12b	100	-0.50	9	1	0	0	16.0	32.0	5,741.5
S13a	100	0	-	-	-	-	-	-	-
S13b	100	-0.50	4	13	0	0	27.2	54.4	-
S14a	100	0	-	-	-	-	-	-	5,533.3
S14b	100	-0.50	0	0	0	0	0.0	0.0	5,404.3
S15a	100	0	-	-	-	-	-	-	5,452.8
S15b	100	-0.50	7	0	0	0	11.2	22.4	5,586.2
S16a	100	0	-	-	-	-	-	-	-
S16b	100	-0.50	9	0	0	0	14.4	28.8	-
S17a	100	0	-	-	-	-	-	-	5,802.4
S17b	100	-0.50	6	8	0	0	22.4	44.8	6,018.6
S18a	100	0	-	-	-	-	-	-	6,031.6
S18b	100	-0.50	9	11	0	0	32.0	64.0	6,322.5
S19a	100	0	-	-	-	-	-	-	5,807.9
S19b	100	-0.50	12	0	0	0	19.2	38.4	5,797.2
S20a	100	0	-	-	-	-	-	-	5,991.9
S20b	100	-0.50	0	7	0	0	11.2	22.4	6,122.6

† Currents, conductance, and average BSA values are reported for combined monomers A and B.
