# Supplementary material for: Complexes of vertebrate TMC1/2 and CIB2/3 proteins form hair-cell mechanotransduction cation channels
Source: eLife. 2025 Jan 8;12:RP89719. doi: 10.7554/eLife.89719 (PMC11709434; doi:10.7554/eLife.89719)
Supplement: Figure 7—source data 1. [file elife-89719-fig7-data1.pdf]

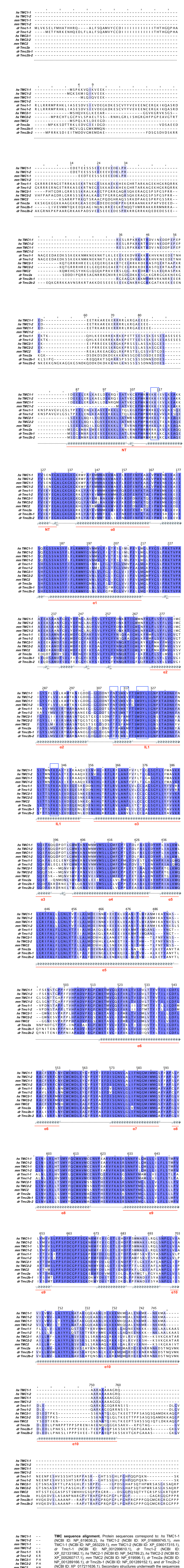

**TMC sequence alignment.** Protein sequences correspond to: *hs* TMC1-1 (NCBI ID: NP_619636.2), *hs* TMC1-2 (NCBI ID: XP_016869745.1), *mm* TMC1-1 (NCBI ID: NP_083229.1), *mm* TMC1-2 (NCBI ID: XP_036017315.1), *dr* Tmc1-1 (NCBI ID: NP_001299610.1), *dr* Tmc1-2 (NCBI ID: XP_021331962.1), *hs* TMC2-1 (NCBI ID: NP_542789.2), *hs* TMC2-2 (NCBI ID: XP_005260717.1), *mm* TMC2 (NCBI ID: NP_619596.1), *dr* Tmc2a (NCBI ID: NP_001289166.1), *dr* Tmc2b-1 (NCBI ID: NP_001289152.1), and *dr* Tmc2b-2 (NCBI ID: XP_017211636.1). Secondary structures underneath the sequences are based on AF2 models of *hs* TMC1. Interface residues between TMC-NT/IL1 and CIB proteins were based on our AF2 model (for NT) and on Liang et al. (blue brackets).
